# Socioeconomic impacts of adolescent pregnancy on education and future employment in Batticaloa District, Sri Lanka

**DOI:** 10.1186/s12889-025-24108-x

**Published:** 2025-10-01

**Authors:** Pramodya Senaratne, Heshan Sameera Kankanam Pathiranage, Dilika Jayawardhana, Kavindu Kaluarachchi, Suhara Gaspe

**Affiliations:** 1 SLIIT Business School, Sri Lanka Institute of Information Technology, Malabe, Sri Lanka; 2 Department of Business Management, SLIIT Business School, Sri Lanka Institute of Information Technology, Malabe, Sri Lanka

**Keywords:** Adolescent Pregnancy, Teenage Mothers, Socioeconomic Status, Early Marriage, Poverty, School Attendance, Labour Market Outcomes, School Dropout

## Abstract

**Background:**

Adolescent pregnancy remains a main concern in Sri Lanka, particularly in the Batticaloa District where the rate is nearly double the national average. Adolescent pregnancy has leads to school dropout and long-term socioeconomic disadvantage for teenage mothers. Because there has been minimal research done within a Sri Lankan context, especially in rural area like Batticaloa, the research aimed to assess how social conditions, accessibility of education, and accessibility of health affect the levels of education and the eventual work opportunities of adolescent mothers.

**Methods:**

A quantitative cross-sectional design was followed with a structured questionnaire being administered among 107 adolescent mothers in Batticaloa who became pregnant between the ages of 15–19 years. The research followed a deductive approach, and data analysis was conducted using Partial Least Squares Structural Equation Modeling (PLS-SEM) with the help of SmartPLS. The model examined the influence of social support, access to education, and access to health on educational attainment and its subsequent influence on labor outcomes.

**Results:**

Findings indicated that low social support, lack of access to education, and lack of access to healthcare significantly derailed the education of adolescent mothers. Level of education was found to be a significant mediator between the three variables and future job opportunities. The majority of the participants had previously dropped out of school by Grade 10, and merely 10.3% were employed, mostly at low-skilled jobs. Analysis confirmed that derailed education directly limits job opportunities and continues to contribute to economic instability among adolescent mothers.

**Conclusion:**

The study brings to the forefront the need for comprehensive, context-sensitive interventions among teen mothers. There has to be reintegration of education, adolescent-friendly healthcare, and vocational training. Reducing stigma and economic and social protection can enhance education and labor market outcomes. Intervention in these domains through a multi-sectoral approach is required to interrupt the inter-generational transmission of poverty and promote the long-term well-being of teen mothers in Batticaloa and similar settings.

**Supplementary Information:**

The online version contains supplementary material available at 10.1186/s12889-025-24108-x.

## Background

Adolescent pregnancy is one of the major issues on impacting education and subsequent socioeconomic outcomes Globally and in Sri Lanka. Educational attainment is the measure for the progress towards SDG Target 4.4, [1] which states as follows:"By 2030, substantially increase the number of youth and adults who have relevant skills, including technical and vocational skills, for employment, decent jobs and entrepreneurship". Many global studies show that in a majority of cases, such as a lack of education leads to high incidences of adolescent pregnancy; education is necessary for equipping girls with the skills and knowledge to postpone early childbearing [2, 3].

Attention from both public and professional individuals towards the controversial social phenomenon of Adolescent Pregnancy has been growing due to the significant impact it has upon long-term wellbeing of young women. According to the World Health Organization [4]"Adolescence is the phase of life between childhood and adulthood, from ages 10 to 19"considered a unique stage of human development. While adolescent pregnancy rate worldwide seems to decline at a slow pace, an estimated 21 million girls aged between 15–19 years in developing regions become pregnant each year, of WHOm more than 50% deliver a baby [5, 6] Similarly reflecting growing concerns regarding Adolescent Pregnancy in Sri Lanka, [7] the 2020 Demographic and Health Survey announced that 30 women out of 1,000 ages 15–19 have begun childbearing in Sri Lanka, highlighting local concerns about this phenomenon. Within South Asian countries, the recorded adolescent pregnancy rate is high in Bangladesh 35% followed by Nepal 21% and India 21% [8] adolescent pregnancy can have significant impact on their level of education, their employment opportunities and marital stability and it increases their financial stability and social dependency on family and neighbors [9].

**Fig. 1:**
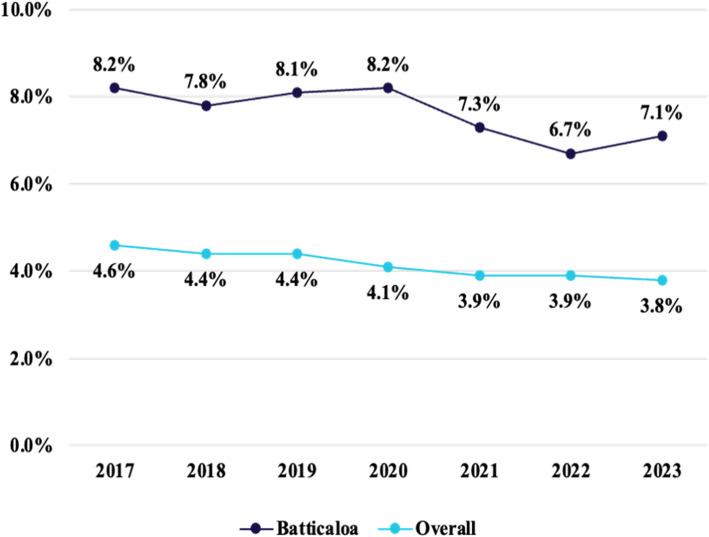
Percentage of Teenage Pregnant Mothers Registered in Batticaloa (2017–2023). Source: Author Illustrated Based on Family Health

Over the last few years, the rate of adolescent pregnancies in Batticaloa has been significantly greater than the national average, triggering a red alert. Family Health Bureau's statistical report indicates that registered pregnancies in Batticaloa which fall under the adolescent category are approximately 8.2%, triggering a serious public concern whereas the national average of the teenage pregnancy is approximately 4% (Fig. 1) [10, 11] This alarming high rate of Batticaloa places it in a prominent position of the adolescent pregnancy problem in Sri Lanka, and it is a significant location for the research.

According to the ministry of education Sri Lanka, school dropout rates statistics of the female students from grades 1–10, Batticaloa with a rate of 1.91 academic years 2020/2021 far above the national average which is 0.72 [12]. Additionally, compared to the overall national average of 76.6% for students passing the G.C.E. A/L examination in 2020, Batticaloa remains low at a passing the exam percentage of 74.2% against 5,781 students [13]. Likewise, considering Sri Lanka's literacy rate, Batticaloa tends to remain below the average rates for the last couple of years. For instance, in 2020 the literacy rate of women in Batticaloa had been reported to be 81.4%, well below the country's national average of 94.4% [14]. These figures point out the lower educational level within the district and position Batticaloa as a priority region to research the effect of adolescent pregnancy on Sri Lanka, especially considering the high rates of adolescent pregnancy.

On the other hand, Department of Census and Statistics revealed that Batticaloa District frequently appears in the statistics of labor with an unemployment rate of high incidence. It was placed in the top 5 districts with the highest level of unemployment during the period of 2017–2019 and was once more noted as the second-highest district in unemployment during the year 2021 [15] Additionally, a study conducted in Anuradhapura, Batticaloa and Colombo reports that barely 9% of pregnant adolescents were so occupied with paid employment as the study was being conducted, also indicating that early motherhood places barriers in their path towards economic activity [16]. Addressing these barriers through targeted interventions, such as improving access to quality education and vocational training, is essential. This study explores global and local findings, demonstrating the impact of education on reducing teenage pregnancies and shaping better employment opportunities for adolescent mothers.

### Problem statement

Adolescent pregnancy remains a serious concern in Sri Lanka. However, as nationwide rates showed a declining trend, certain areas such as Batticaloa are continuing to experience higher incidence. Adolescent mothers in these regions face major issue in the education sector and have inexperienced labor market access. Contrary to studies around the world that highlight the effects of this issue on adolescent mothers, there exists a gap in studies targeted toward the Sri Lankan context with these all factors together.

While global studies point to effects of adolescent pregnancy in relation to educational attainment and employment prospects, there is lack of in-depth research focusing on Sri Lanka. Studying the impact of adolescent pregnancy on young mothers'education and their future employment is key to developing relevant interventions and policies fit to the unique socio cultural and economic circumstances of the country. addressing this gap would empower the affected while adding to the overall development of society.

### Research aim and objectives

This study's main aim is to analyze the impact of adolescent pregnancy on educational attainment and future employment opportunities of adolescent mothers in Sri Lanka, with a special emphasis on findings especially from the Batticaloa District. The study has tried to consider socioeconomic status, access to education, and access to healthcare as variables in the educational achievement of teenage mothers. Secondary objectives are a number of specific research questions: measuring for social factors affecting educational attainment of adolescent mothers, assessing the level of influence of access to education on their academic progress, evaluating the impact of healthcare access on their educational outcomes, and lastly analyzing the effect of educational attainment by adolescent mothers on employment opportunities.

### Theoretical framework: understanding adolescent pregnancy and educational outcomes

This research will look into the social aspects and personal and social causes behind the effects of adolescent pregnancy. To explain what leads to adolescent pregnancy, researchers have included in their conceptual model the two main theories, Social Learning Theory and the Socio-Ecological Model. In Sri Lanka, this approach highlights that cultural pressures, economic problems, and hard rules play major roles in limiting the education and job options for adolescent mothers.

The Ecological System Theory (1979) by Bronfenbrenner outlines the ways different systems in the environment can influence adolescent pregnant mothers [17]. For these girls, it is mostly their family, school, and peer group environments that play a major role from the outside. According to [18], there are more chances of dropout for adolescent mothers in Sri Lanka because of family issues and unpleasant events at school. As Bandura points out in her [19] social learning theory, people learn the behavior they show by observing and copying others, and by being reinforced for their behavior.

First, modeling explains how adolescents are apt to emulate behaviors observed in role models. When early motherhood in the immediate social context is observed, it is a behavior that is learned and sanctioned by society [19] Second, reinforcement focuses on the difference between positive and negative roles. The cultures that accept or even recognize adolescent pregnancies continue to encourage such behavior to render it less rewarding to avoid early parenthood [20] Third, self-efficacy is applicable in decision-making. A low self-efficacy decision-making mechanism, triggered by socio-economic limitations and limited education goals, gives a weaker adaptive agency to an adolescent to avoid teenage pregnancy or undertake long-term goal-oriented action towards education goals [21] Social gender roles in Sri Lanka can be used to reinforce the perception that early marriage and motherhood are ideal or normal experiences for young women and therefore continue to normalize adolescent pregnancy [22].

### Impact of adolescent pregnancy on educational attainment

Some pregnant adolescents and mothers experience challenges balancing parenting responsibilities with schooling [23–25] hence the massive number of dropouts. United Nations Educational, Scientific and Cultural Organization reveal that this gender disparity in school completion rates is based on pregnancy-related school dropout rates, which has largely contributed to this ambiguity [26] For instance, Gender gaps in education [27] showed that 90 out of 126 countries show a lag of women's educational attainment compared to men, thereby illustrating the glaring disparity in educational equity on a global scale. While Adangabe 2020, Narita R 2016 and Naidoo J 2021, [23, 28, 29] argue that motherhood at a very young age correlates negatively with a girl's educational attainment and that this contributes to securing zero gender inequality in education, the situation is aggravated in low-middle-income countries (LMICs) [30], where every year, over 12 million adolescent girls aged 15 to 19 become mothers, impacting both educational and economic achievements adversely [31, 32].

Being a mother and student juggling extra responsibilities and challenges on adolescent mothers [33]. Not managing to keep a balance between the two results in concentration and absenteeism by students and sometimes even a re-dropout from school [34]. Disregarding the views of teenage mothers as well as their suffering in striking challenges in the form of childcare, financial difficulties, and the absence of social support renders the implementation of the school re-entry policy less effective. It will thus remain, as it were, a mere bureaucratic law pregnant with no positive effect on the lives of teenage mothers [33].

According to Adolescent Pregnancy Fact Sheet 2020 [35] and UNICEF 2024 [36] At a yearly rate, over 16 million girls aged 15 to 19 and one million under age of 15 years old give birth, with 41.6% dropping out of school due to pregnancy: More than six million girls who are either pregnant or already parenting are out of school [35–37] Therefore, teenage pregnancy presents a big reason for dropping out of school among basic school adolescents (Adam et al., 2016); the challenges that occur when the girl transitions into adolescent motherhood are intensified by a lack of parental and social support [38, 39] These difficulties act as barriers to the education of adolescent mothers and lack of policy interventions. To overcome educational barriers, a school re-entry process has been set up that allows pregnant girls to attend school once they have given birth [31, 32]. Existing studies indicate stigma and discrimination as barriers to the school re-entry of adolescent mothers [40–42] However, there is currently an evident lack of comprehensive literature that considers how those challenges are tackled by schools and the ensuing effects of future employment.

### Impact of social factors on adolescent mothers’ education

The socio-economic status of the family influences adolescent pregnancy. Acharya Dev Raj investigated that [43] in a research on determinants of adolescent pregnancy in South Asia, found that adolescents WHO belonged to middle or poor families have a high chance of getting pregnant as opposed to those who belonged to rich families. The parents are able to marry off their daughters at an early age because of poverty, and these early marriages have a very strong association with adolescent pregnancies, In 2015 Pregnancy among Unmarried Young Women in Urban Kenya [44] confirms this fact that the economic status of the family determines the age at first marriage especially for poor families and this is true because if a family is poor, they can decide to marry off their daughters at an early age so that they can get bride price and this can result in teenage pregnancies.

Socio-economic status, education level, cultural determinant and family constellation were identified to be risk factors for adolescent pregnancy in South Asia. Through the use of a retrospective questionnaire, [45] in Rural Nepal established that the prevalence of adolescent pregnancy is tremendously high in lower social classes (52%) than the higher social classes (26%). Hindu adolescents were also more likely to become pregnant (p < 0.001) than Buddhist adolescents. Social and structural disadvantage, poverty and gender all combined to make young people extremely vulnerable to teen pregnancy [46].

Social factors significantly affects the educational and future career of adolescent mothers around the world, including that of Sri Lanka. In most cases, those adolescent mothers from disadvantaged backgrounds face serious barriers on their way to continued education because of factors such as resource access, child care, and child care support, and stigma on them from society on becoming pregnant [47]. In 2013, [48] corroborated the hypothesis stating that social pressure is directly linked to teenage pregnancy. They pursued their investigations in three districts of Sri Lanka, such as Anuradhapura, Batticaloa, and Colombo; the sample comprised 510 teenage mothers. The study was set to identify the risk factors affecting teenage pregnancy in Sri Lanka. According to the research, factors such as personal characteristics, low educational attainment, poor parental supervision, and poor support from teachers comprised certain risk factors for teenage pregnancy.

Apart from this other researchers have also confirmed that teenage pregnancy has an associative relationship with ethnicity, social, and cultural variables. In fact, especially in many developing countries, minority communities tend to have a higher number of teenage mothers in them, and such reasons are being ignored by dominant groups, economic backdrop, and their ways of life, etc. [20].

In Nigeria, for instance, financial constraints of low-income families have been associated with increased rates of school dropouts among pregnant adolescents, [49] while stigma and insufficient institutional support are the additional obstacles encountered in Namibia's Zambezi region [50]. Therefore, that is the intersection of the economic milieu with the societal one into which restrictive factors create an environment for educational progress to young mothers.

Marriage and family arrangements aggravate educational challenges for adolescent mothers. Studies indicate that adolescent mothers living in non-supportive marriage arrangements, or without extended family support, were 73% less likely to complete secondary education than their counterparts living with both parents [51]. Emerging concerns and case studies on child marriage emphasize that these trends are very much play in the Sri Lankan context, where early marriages and cultural expectations often limit opportunities for educational reentry [52]. To effectively address the above-mentioned socio-economic challenges, a more multifaceted approach is needed that investigates enhancing parental engagement, community support systems, and policy-driven initiatives to strengthen the accessibility of education and economic opportunities offered to adolescent mothers.

### Impact of access to education on adolescent mothers’ education achievement

Education is a universal tool for mitigating the negative effects of adolescent pregnancy, which has commonly resulted in interruption to education and subsequently limited future opportunities. In Developing countries like Malawi and Rural Lao, 17% of girls drop out due to pregnancy, and less than 9% return to school, which interrupts not only their education but also their socio-economic opportunities [53, 54]. In the same way, the Organization for Economic Co-operation and Development (OECD) countries records that school-leaving rates for women aged 18–19 fall by 30% among those with children compared to their peers [55].

According to the WHO [4] and UNFPA [56] Globally, 16 million adolescent girls give birth annually, with many unable to return to school due to stigma, low self-esteem, and financial constraints [57, 58]. Adolescent pregnancy is linked to inconsistent attendance and poor academic performance in South Africa [59], while U.S. states with abstinence-only education see higher pregnancy rates, emphasizing the need for comprehensive sex education [60].

In Chille, adolescent motherhood reduces high school attendance by 24–37% [61], while in Ghana’s Kpando Municipality, 79% of Adolescent mothers drop out due to poverty and childcare challenges [24]. New Zealand’s longitudinal study shows a 39.6% dropout rate among young mothers, driven by academic and familial factors [62, 63]. In Namibia’s Zambezi Region, stigmatization and lack of institutional support exacerbate school dropouts [50], while in Nigeria, 1.3 million girls leave school annually due to early motherhood pressures [64]. These findings highlight the urgent need for policies addressing educational access, comprehensive sex education, and supportive systems for adolescent mothers.

In Sri Lanka, particularly in Batticaloa, 8.4% in 2020 adolescent pregnancy rates correlate heavily with barriers to education [65] Finding from Rajarata Pregnancy cohort in 2021 show that the higher education level of the mother significantly less likely to experience unintended pregnancies [22]. Some of the contributory factors are poverty, lack of educational facilities, and the absence of sexual education in curricula, which would allow for the young mothers to return to school [65]. Addressing these challenges through Awareness campaigns and reproductive health education should be instituted early in order to prevent adolescent pregnancies and improve educational performance [66]. The same confirms the international evidence in the sense of education in also battling the socio-economic inequalities caused by adolescent pregnancy.

### Impact of access to healthcare on adolescent mothers’ education attainment

Access to healthcare has proven instrumental in reducing the adverse effects of adolescent pregnancies in the global as well as Sri Lankan context. Pregnancy among adolescents is spoken about as something less dangerous, compared to other maternal health aspects, because it is culturally accepted along with family support in Sri Lanka [67]. Adolescent pregnancies globally are faced with precarious health, education, and socioeconomic challenges-poor physical and mental health up until reduced educational attainment and highest risks of poverty that linger until midlife [68–70]. Limited access to health care worsens these outcomes with poor prenatal and post-maternal care, which directly affects maternal and child health and are intertwined with stigma and societal rejection [64, 71]. Adolescents in sub-Saharan Africa, low levels of family planning use and insufficient sexual and reproductive health knowledge usually means the girl faces unnecessary health risks [53]. Thus, there is an urgent need for focused interventions that combine preventive and supportive approaches, including making health services accessible and developing community-based awareness programs.

Access to healthcare and eradicating stigma has been seen to be effective in supporting the health and well-being of adolescent mothers [72, 73]. Comprehensive family planning and early access to sexual and reproductive health education have been shown to improve health outcomes globally [74, 75]. The reduction of health risk and betterment of life trajectories of adolescent mothers across the globe are very much dependent on comprehensive healthcare access and education dealing with these gaps.

Systemic healthcare interventions, such as the Family Health Bureau and Adolescent and Youth-Friendly Health Services (AYFHS), established in 2005, have enhanced care for adolescent mothers through clinic and field-based services [11]. Additionally, pre-conception clinics and health education sessions for newlyweds address risks and promote maternal care [11, 22]. These integrated efforts highlight the importance of systemic health and education solutions in improving reproductive health and reducing socio-economic impacts. Adolescent mothers are vulnerable to constraints in accessing healthcare services because of stigma, lack of confidentiality, and not having of adolescent-friendly services [76] In Nepal and Bangladesh, among other countries, evidence shows the need to introduce adolescent-specific reproductive health services within the primary care system to ensure better access and less stigma [77] Enforcing these systems is very important in supporting the well-being and long-term results of adolescent mothers.

### Impact of adolescent pregnancy on future employment opportunities

#### Income disparities

Globally, the link between adolescent motherhood and income disparities is well-documented. Early pregnancies disrupt education, limiting young mothers’ access to higher-paying jobs that require specialized skills or advanced qualifications. Evidence from Brazilian data [28] emphasized that teenage pregnancy reduces opportunities for higher learning, leading to a cycle of economic insecurity and poverty. Similarly, Almeida and Aquino (2011) in Brazil [78] showed that adolescent mothers often face increased dropout rates and reduced access to stable employment, leaving them reliant on informal, low-paying jobs with limited benefits.

Moreover, Young women adult in chillie [79] identified that young mothers frequently resort to unskilled or semi-skilled labor, which constrains their career advancement and lifetime earnings. This pattern is quite indeed more pronounced in low-income and developing countries where social support systems and economic support are poorly developed.

Similarly, in Sri Lanka, there is very much evident, particularly in Batticaloa. Because of the country's relatively strong social indicators in education and healthcare, entering adolescent mothers face substantial barriers in re-entering the labor market. The limited availability of skill training and childcare services exacerbate these problems such that younger mothers end up in precarious low-paying jobs [80]. Evidence shows that financial strains are usually faced by adolescent mothers, and this is worsened by societal stigma, which limits them further from pursuing any formal education or stable employment [81]. This is what has been established globally; such mothers have limited opportunities of getting hired in jobs that would allow such growth over the long haul or financial security, usually preserving income inequality.

#### Relationships and family dynamics

The family background and early relationships significantly affect the employment achievements of adolescent mothers. Studies show that adolescent pregnancies usually lead to early marriage or unstable partnerships, which severely limit educational and professional opportunities. In 2002 Northern Ireland [82] found that those who marry at an early age were more likely to drop out of school than their non-adolescent mothers, thus obtaining fewer qualifications. In this way, they are shut out to skilled jobs and continue to rely on basic unskilled labor. In additionally, Data from Manitoba [83] demonstrated that adolescent, young mothers in Canada are regularly on income support programs due to limited employment opportunities; this pattern is also reflected in various contexts worldwide.

In Sri Lanka, early unions and traditional gender roles reinforce and restrict young mothers in gaining access to education and employment. The Batticaloa district exemplifies this, where cultural norms often prioritize domestic responsibilities over professional aspirations for women. United Nation Children’s Fund 2024 [57] reports that one in four women in South Asia, including Sri Lanka, marry before the age of 18, which curtails their ability to pursue education and develop professional skills. Also Young Mexican mothers [84] noted that cultural and familial stressors, such as the stigma associated with young motherhood, significantly lower self-esteem and hinder the pursuit of aspirational employment. The lack of adequate childcare and support systems within families further exacerbates these challenges, forcing many adolescent mothers to future career opportunities in favor of caregiving responsibilities.

By offering a region-specific analysis of the effects of adolescent pregnancy on education and future career prospects in Batticaloa District, Sri Lanka, this study addresses in the identified research gaps. It provides a quantitative evaluation of the various ways in which healthcare access, educational access, and economic status affect adolescent mothers'educational and career outcomes. The study contributes to policy discussion on enhancing educational reintegration and vocational training programs by combining local findings with international knowledge.

### Data and methodology

#### Introduction

Adolescent pregnancy is held to be a serious social problem in Sri Lanka, especially with the educational background and work opportunities of the adolescent mothers taken into consideration. The main purpose of this research project is to study the consequences of teenage pregnancy on education and future employment opportunities in the Batticaloa District in Sri Lanka. The chapter describes the research philosophy, approach, and design employed in the research. Further, it explains the strategies used to select the sample, the data-collection techniques used, and the methods used in analyzing the data-to attain the objectives of the research study.

**Fig. 2:**
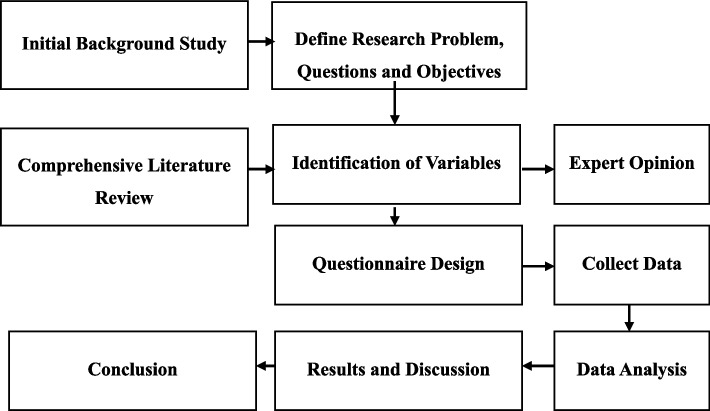
Research flow diagram. Source: Author’s compilation

As illustrated in Fig. 2, a structured, systematic approach was used in the study, employing the waterfall method passing through distinct, sequential phases. Each step of the research was given due consideration before moving forward to the next stage, providing for a clearly defined sequence starting with the literature review through to data collection and analysis.

In a research methodology, multiple quantitative techniques are combined to study a problem from several perspectives. This begins with an in-depth literature review on the subject around which the study is framed and the key variables and theoretical gaps circumscribed. From this foundation, a conceptual framework is built that directs the designing of a structured questionnaire to collect relevant data from teenage mothers in the study area.

Data collection was carried out by way of the face-to-face method of administering a questionnaire and the responses were analyzed though Smart PLS. The analysis was aimed at testing the study's hypotheses and examining the relationships between adolescent pregnancy, educational attainment, and future employment opportunities. The methodology seeks to generate actionable insights toward policy recommendations, social support interventions, future and present research efforts on adolescent pregnancy in Sri Lanka.

#### Conceptual framework and hypotheses

The negative effects of adolescent pregnancy not only impact on the level of education attained by pregnant adolescent mothers but generates a subsequential effect on their employment prospect. Research conducted by the World Bank highlights that teenage mothers are less likely to continue their education after the pregnancy, drawing a limit to their economic opportunities and perpetuating cycles of poverty [85]. Besides, as noted earlier, past research has shown that variables such as socioeconomic status including family income and social standing, and availability of education and healthcare, are vital in influencing the educational attainment of adolescent mothers.

**Fig. 3:**
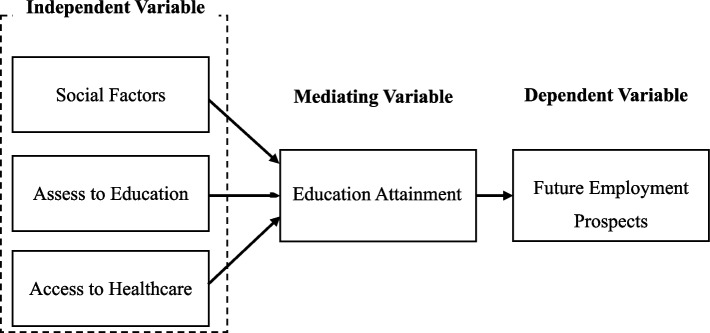
Conceptual framework. Source: Author illustrated based on literature review

**Fig. 4:**
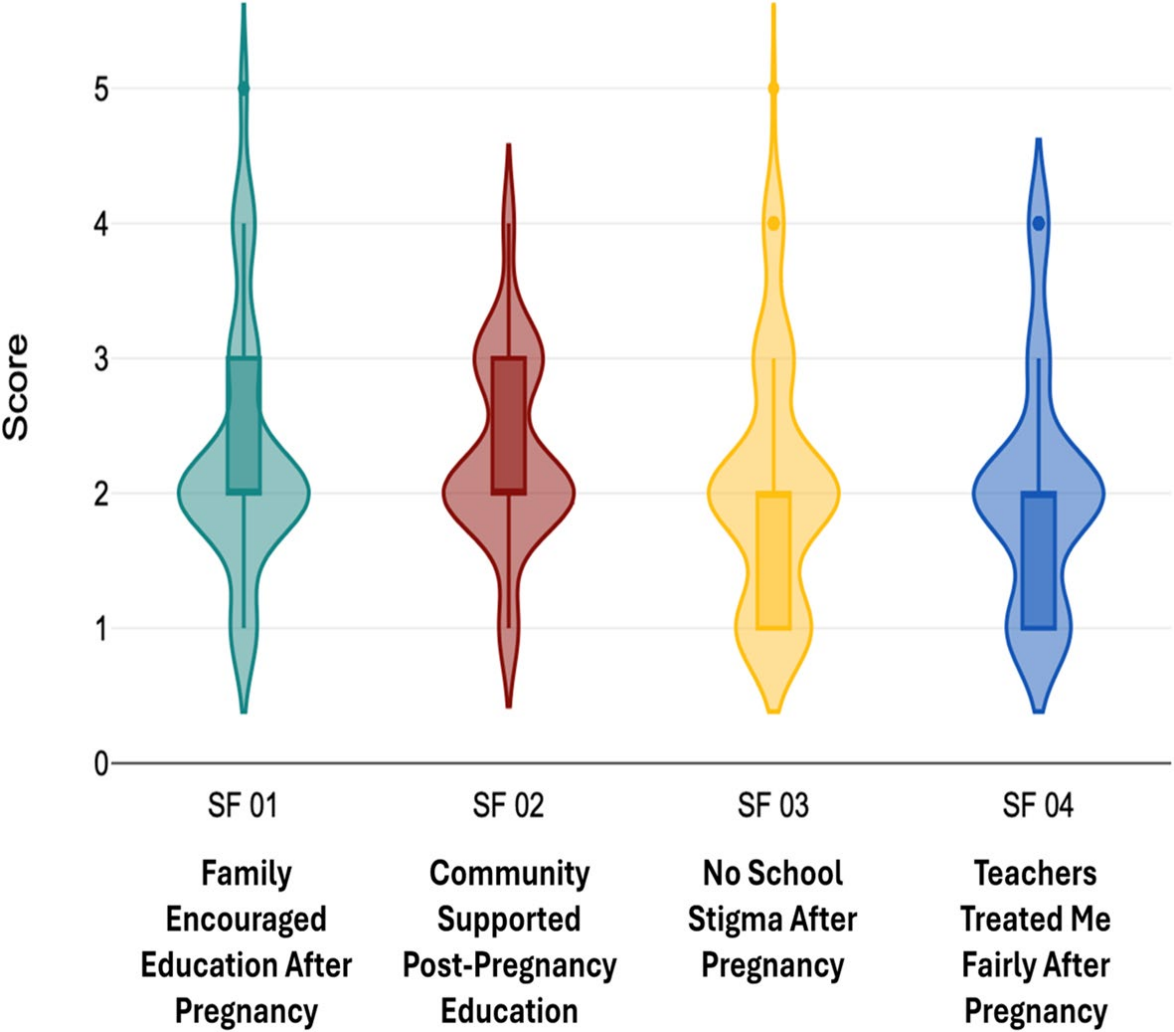
Distribution of responses on social factors. Source: Author illustrated based on primary data

**Fig. 5:**
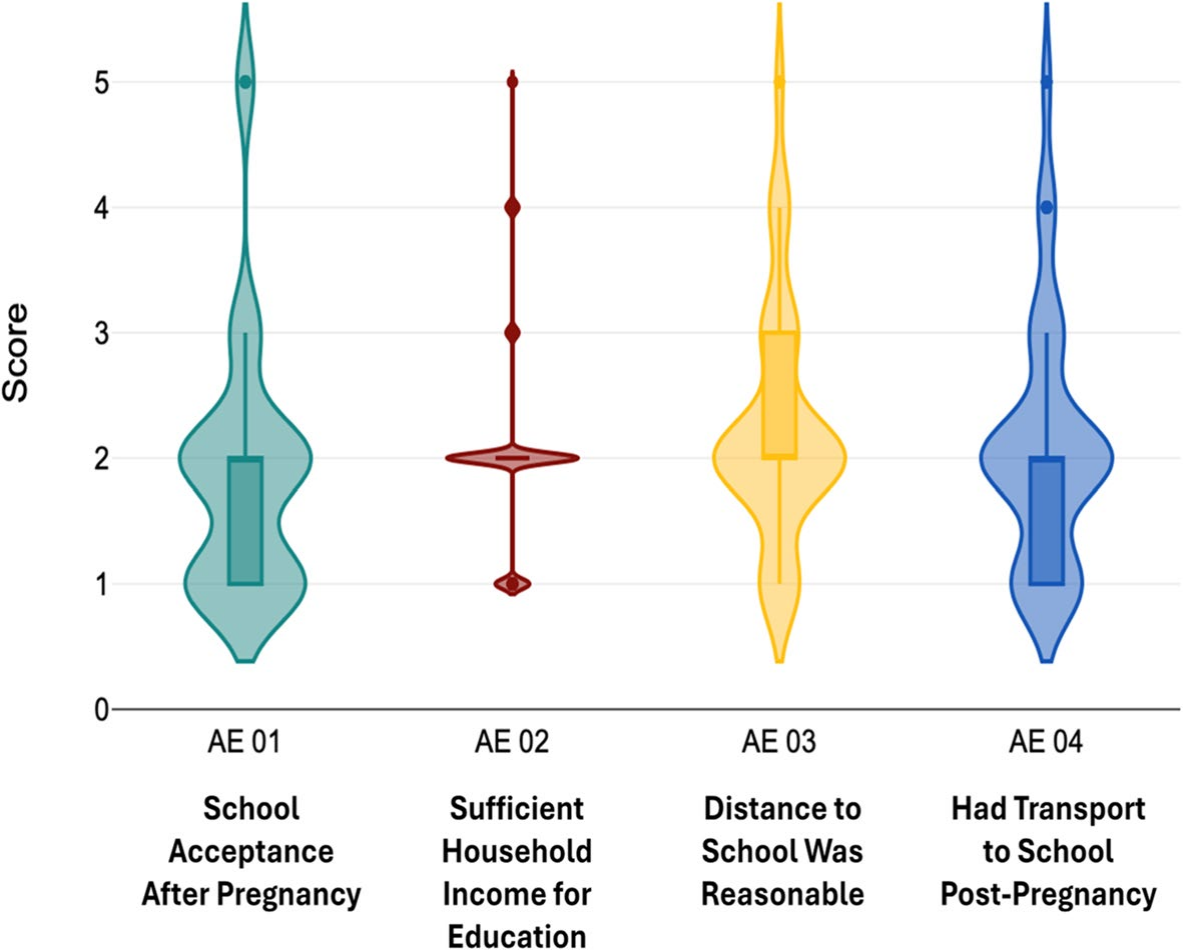
Distribution of responses on access to education. Source: Author illustrated based on primary data

**Fig. 6:**
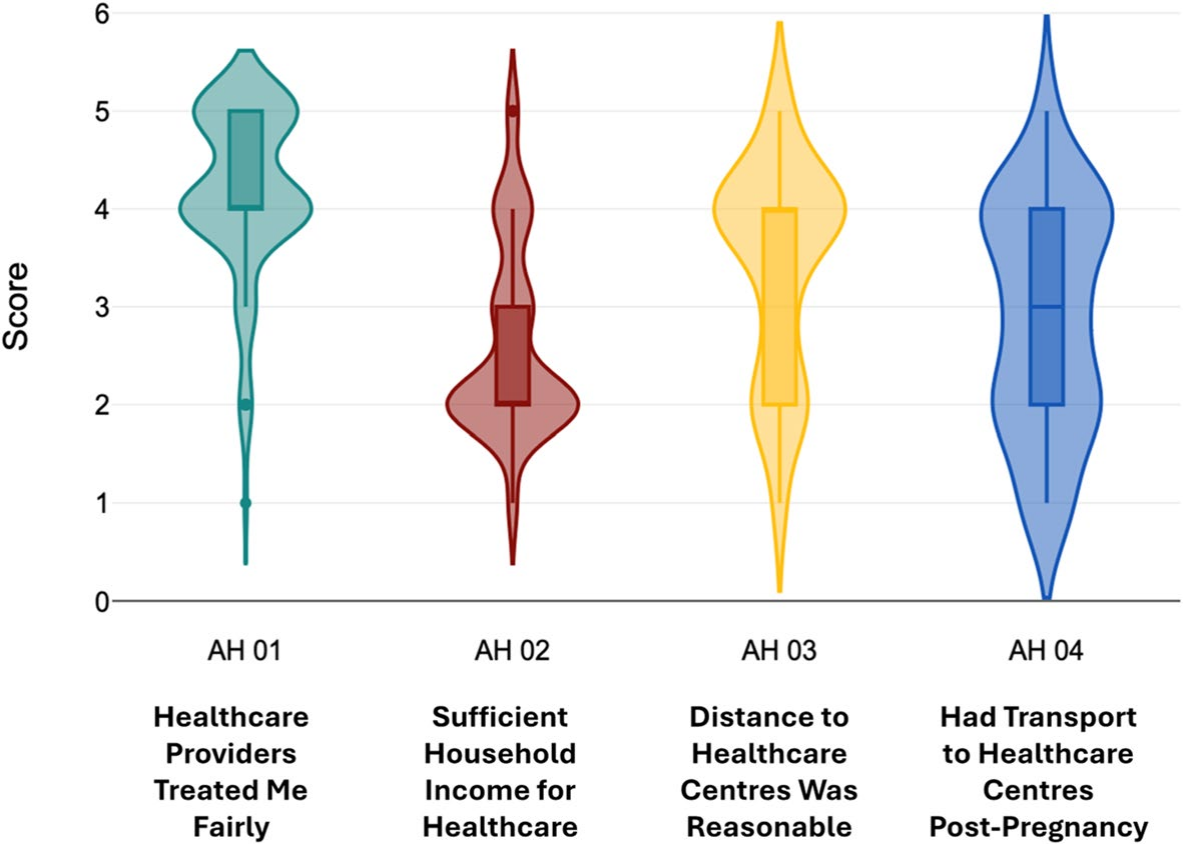
Distribution of responses on access to healthcare. Source: Author illustrated based on primary data

**Fig. 7:**
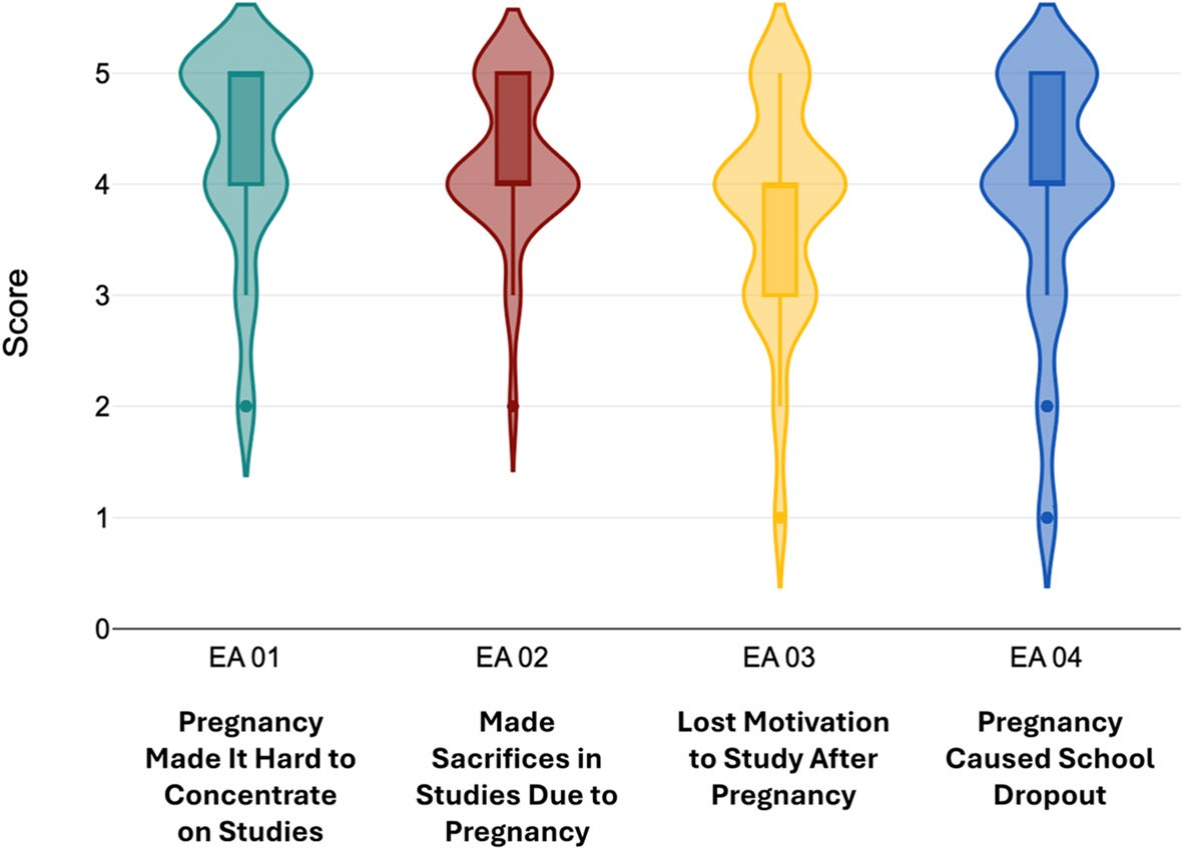
Distribution of responses on educational attainment. Source: Author illustrated based on primary data

**Fig. 8:**
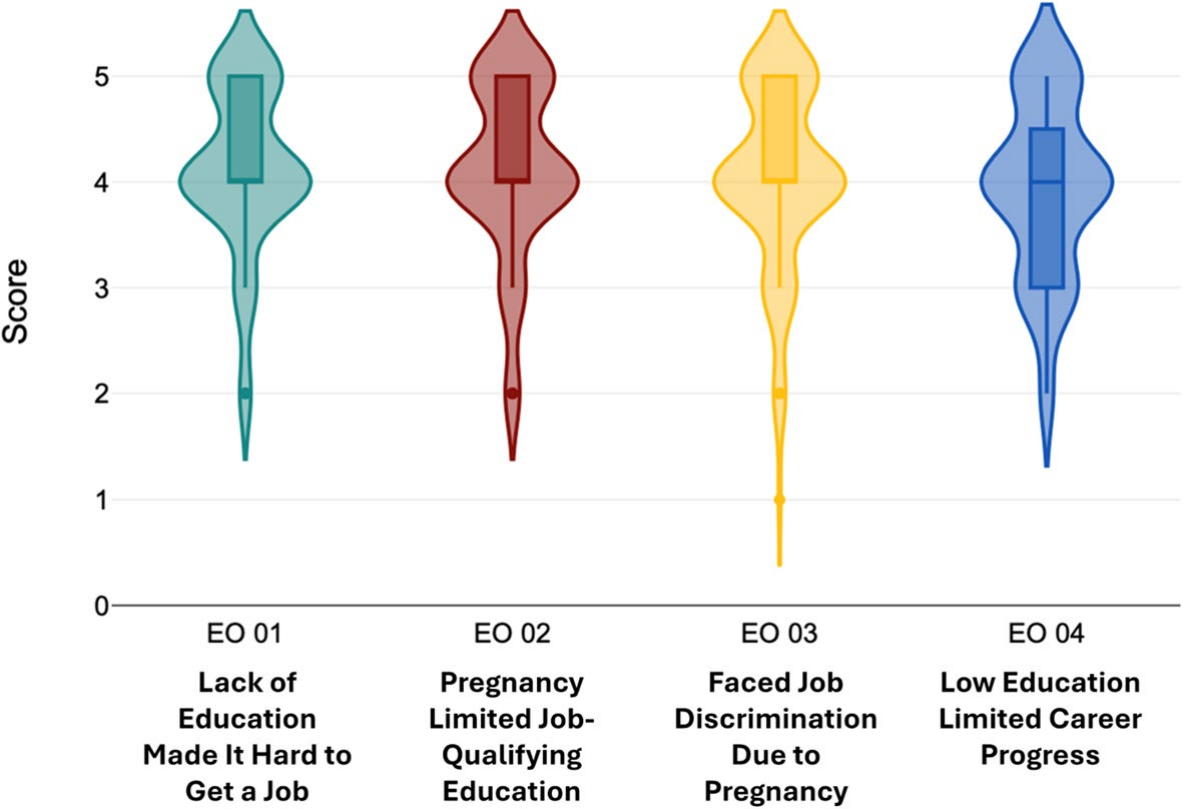
Distribution of responses on employment outcomes. Source: Author illustrated based on primary data

During an extensive literature review, many connections were noticed between those factors which disrupt the education of adolescent mothers after pregnancy and the consequent effect on employment. To envision the relation of these factors more clearly, the above illustrative conceptual framework was developed. Figure 3 highlights multifactor problems that need to be tackled within the confines of this research. In alignment with this objective, the independent variables of this study are Socioeconomic Factors, Access to education, and Access to healthcare, while future employment outcomes are considered the dependent variable Figs. 4, 5, 6, 7 and 8.

To improve the strength of results, mediating variables can be used for supporting the independent and the dependent variables. Educational attainment is one of the mediate variables. Thus, in the framework, the mediate variable influences both the dependent and independent variables. Hence, the dependent variable, Future Employment, would be directly affected by the educational attainment of an adolescent. It emphasizes the impact of these variables, implying that the level of education attained by pregnant adolescents contrasts not only with their immediate academic achievement but also with their long- term impact of employment opportunities and payment for economic sustenance. Based on the connections established through the conceptual framework and literature the following hypotheses was developed:

- H1: *There is a significant relationship between social factors and educational attainment.*
- H2: *There is a significant relationship between access to education and education attainment.*
- H3: *There is a significant relationship between access to healthcare and education attainment.*
- H4: *There is a significant relationship between educational attainment and future employment.*

### Data

#### Research design

Considering the research objectives and questions that a quantitative research design is the more suitable method due to the objectivist nature of this study and its intense focus on cause-and-effect relationships between clearly specified variables. The study adopts a cross-sectional deductive approach to research [86–88] This allows analyzing the inducing factors such as social factors, access to education, access to healthcare as independent variables, with future employment opportunities of adolescents in Batticaloa district, Sri Lanka as dependent variables. The relationship is further mediated through educational status, thus allowing the study to examine interaction effects within the model.

#### Sampling design

For this study, researchers utilized the snowballing method to recruit participants. In snowball sampling, which is a non-probability sampling method, subject participants are asked to recruit further participants from anatomy of acquaintances. It is cited to be more effective for hidden or hard-to-reach populations or for investigations of personal experiences that are sensitive [89]. Adolescent mothers are one such population, normally subjected to social stigma without consideration of their feelings, and sometimes with serious underreporting, rendering random sampling impractical.

According to Sadler and Swartz [90] adolescents with early motherhood may feel unwilling to disclose their condition because of the fear of judgment or mistrust of official systems. Having a snowball referral allowed trust to build from peer referral, thus making for safer and more comfortable participation.

Existing literature such a [91] has pre-established snowball sampling as a viable method of obtaining credible community information from adolescent pregnancy or other low-resource, socially conservative, or sensitive populations. There is therefore not just an appreciation of such a strategy as ethical, but as a design that is well-suited to obtaining credible information from a marginalized population on any official sampling procedure.

#### Method of data collection

The population of this research was comprised of all adolescent pregnancy mothers in the Batticaloa District of Sri Lanka. The SLIIT Business School and the SLIIT ethical review board both examined and approved this study. As of June 2024, 150 pregnant mothers under the age of 19 had been registered for family planning consultations in the Batticaloa District [92] According to the, [93], the required sample size for this group was determined to be 108. The researchers were able to acquire a total of 109 data points.

The study populations were women WHO had been pregnant between the ages of 15 to 19 years. Although some of the participants were between age 20 and 25 years old at the time of data collection, all of them had been pregnant as adolescents, aligning with the study’s focus. The age of pregnancy was verified to be prior to the 20th birthday from cross-checking the information on the period of clinic records with the age. Snowball sampling technique was employed in choosing participants [94]. Here, volunteers who were eligible for participation were first selected and then asked to bring in others from their networks. This approach permitted participants from hard-to-reach or hidden populations to be recruited, with the possibility of maintaining practicality as well as efficiency in the data collection process.

#### Questionnaire design

The questionnaire was designed based on the research goals and conceptual framework of the research utilizing existing models as established in previous studies mentioned in the literature review. Even though the questionnaire has been initially developed in the English language, it was subsequently translated into the Tamil language for better understanding by the respondents. We followed [95] approach by using the technique of back translation in order to achieve linguistic equivalence between the two versions. Data were collected by experienced interviewers who were native speakers of the relevant mother tongue of the respondent. It consisted of six sections: one covering demographic details, and sections two to six having questions intended to measure attitudes and perceptions.

All questions were scored on a 5-point Likert scale ranging from"Strongly Disagree"(1) to"Strongly Agree"(5). Marital status, ethnicity, and residence were also asked through other questions. These questions and measurements are presented in the Table 1. Data collection took place during mid-April to end of May 2025 in Batticaloa District. Cross-checked data were gathered for validation and reliability purposes to confirm the integrity of the primary data.

**Table 1:** Operationalization of research variables

Type of Variables	Variables	Indicator	Measurement	Question Items	Source
**Independent** **Variables**	Social Factors	SF 01: Family Encouragement	1: Strongly Disagree 2: Disagree 3: Neutral 4: Agree 5: Strongly Agree	Q8-Q11	[96]
		SF 02:Community Support			[97]
		SF 03: Stigma or Discrimination			[97]
		SF 04: Treatment from Teachers			[97]
	Access to Education	AE 01: School Acceptance	1: Strongly Disagree 2: Disagree 3: Neutral 4: Agree 5: Strongly Agree	Q12-Q15	[98]
		AE 02: Sufficient Financial Support			[99]
		AE 03: Proximity of School			[99]
		AE 04: Transportation to School			[99]
	Access to Healthcare	AH 01: Respectful Maternity Care	1: Strongly Disagree 2: Disagree 3: Neutral 4: Agree 5: Strongly Agree	Q16-Q19	[100]
		AH 02: Sufficient Financial Support			[101]
		AH 03: Proximity of Healthcare Services			[101]
		AH 04: Transportation to Healthcare			[100]
**Mediator Variable**	Educational Attainment	EA 01: Study Disruption Due to Pregnancy	1: Strongly Disagree 2: Disagree 3: Neutral 4: Agree 5: Strongly Agree	Q20-Q23	[64]
		EA 02: Educational Sacrifices			[102]
		EA 03: Decrease in Educational Motivation			[103]
		EA 04: School Dropout			[98]
**Dependent Variable**	Employment Outcomes	EO 01: Employment Barriers	1: Strongly Disagree 2: Disagree 3: Neutral 4: Agree 5: Strongly Agree	Q24-Q27	[104]
		EO 02: Education Completion Difficulties			[104]
		EO 03: Employment Discrimination			[104]
		EO 04: Limited Career Progression			[104]

### Methodology

This study employs a positivism research philosophy to examine the complicated phenomenon of adolescent pregnancy by collecting primary data through questionnaires and approaches to attain a wider perspective of its impacts [105, 106]. Quantitative analysis is then employed in order to examine whether there is any cause-and-effect relationship between adolescent pregnancy, education, and employment opportunities in Batticaloa district. Specifically, the analysis was carried out via Partial Least Squares Structural Equation Modeling (PLS-SEM) according to Smart-PLS software.

#### Method of data analysis

##### Convergent validity

Convergent validity was determined by checking the factor loadings of items on their corresponding constructs. According to Anderson (1989) [107] convergent validity is considered manifest when the indicators load sufficiently into their respective latent variables. SmartPLS 4.0 was used to conduct the second-order Confirmatory Factor Analysis (CFA) for the purpose of assessing convergent validity of the measurement model.

It was a five-construct CFA model that incorporated the following constructs: social factors, access to education and access to healthcare as independent variables, educational attainment as a mediator, and lastly, future employment prospects as the dependent variable. All constructs are reflective in modeling. The standardized factor loadings were more than the minimum acceptable threshold of 0.7, and they were statistically significant (*p* < 0.05).

##### Reliability and validity test

In this study, the internal consistency of the constructs was assessed using Cronbach’s alpha. The range of item loadings and corresponding Cronbach’s alpha values for the construct’s social factors, access to education, access to healthcare, educational attainment, and future employment prospects are given in Table 3. These results show a high level of internal consistency for each construct thereby confirming the reliability of the measurement scales used in this study.

Validity is established according to the relevant analysis so that whatever is derived through any measuring tool can be correctly interpreted. To assess validity means setting up whether the scale items quantify precisely what has to be quantified for the purpose of achieving the aims of the study. Establishing the validity is, however, more challenging than establishing the reliability of the measuring instrument.

##### Discriminant validity

Discriminant validity, as defined by Gilbert, Churchill J 1979 [111], is the extent to which it is thought that two measures of different constructs are distinct. Study discriminant validity was determined through inter construct correlations [109] and by employing the comparison of the square root of the Average Variance Extracted (AVE) for every construct with its correlation with other constructs [112]. All of the correlations among the social factors, access to education, access to healthcare, educational attainment, and future employment prospects, as shown in table iii, were all below the cut-off value of 0.80 that was suggested. Furthermore, the square root of the AVE for all the constructs was higher than the highest correlation of the construct with any other construct. Therefore, these findings demonstrate that each construct uniquely represents a dimension of the model, thus attaining discriminant validity.

##### Structural equation model

It has been validated using Partial Least Squares Structural Equation Modeling (PLS-SEM), with the aid of SmartPLS software, according to [113]. It assists the study in analyzing intricate relationships between the latent variables. The SEM method support to ascertain the structural influence pathways, model latent constructs, and statistically validate the theoretical postulates introduced within the in the conceptual framework.

This constructed graphical model with SmartPLS will display structural relationships between the variables graphically (see Table 2). Path coefficients were estimated to find out the strength and direction of the relationships, while the hypothesized paths were tested through bootstrapping techniques providing t-statistics for testing each assumed path's significance.

In addition, PLS-SEM may even be employed using a moderate sample size when robustness had been assured. Its usability is equally matched by the practical significance of its application to predict human behavior in social scientific research, for instance, in the present situation where moderating effects of education level are taken into account [114]. Therefore, such advantages notwithstanding, PLS-SEM is chosen to study the model and test all hypothesized relationships adequately.

**Table 2:** Characteristics of respondents

n = 107	**Count**	**Percentage**
**Age at the Time of the Study**		
10 to 18	66	61.7%
18 +	41	38.3%
**Age at the Time of the Pregnancy**		
10 to 15	12	11.2%
16 to 19	95	88.8%
**Ethnicity**		
Tamil	58	54.2%
Muslim	32	29.9%
Sinhalese	17	15.9%
**Marital Status**		
Married	74	69.2%
Single	24	22.4%
Divorced/Separated	9	8.4%
**Level of Education**		
No formal education	1	0.9%
Grade 1–5	1	0.9%
Grade 6–10	52	48.6%
GCE Ordinary Level	47	43.9%
GCE Advanced Level	6	5.6%
**Employment Status**		
Unemployed	96	89.7%
Employed	11	10.3%
**Occupation**		
No job	96	89.7%
Retail Sales	5	4.7%
Garment Worker	5	4.7%
Healthcare Worker	1	0.9%

## Results

### Background information on results and discussion

As explained in the previous sections, a questionnaire was used to collect data from adolescent mothers in the study focus area. 109 responses were collected initially and stored in Excel for cleaning procedure. While doing the cleaning procedure, 2 responses were rejected due to missing data. The rest of the 107 responses were analyzed using Smart-PLS software to obtain the results indicated in this section.

#### Characteristics of respondents

Among 107 adolescent mothers WHO had been interviewed, 61.7% were found age between 10 and 18 years old, while 38.3% were above 18 years old, with a mean age of 18 years. At the time of the pregnancy, 11.2% of the participants were between the ages of 10 and 15 years, and 88.8% were between 16 and 19 years of age. Average age at the time of the pregnancy was 17 years. Ethnic division showed that 54.2% of the teenage mothers participating were Tamil, followed by 29.9% Muslims and 15.9% Sinhalese.

With regard to the marital status at the time of conducting the study, 69.2% were married, 22.4% were single and 8.4% divorced or separated. According to the Sri Lankan Demographic and Health Survey in 2016 [115] early and child marriages are very prevalent amongst the Sinhalese, Tamil, and Muslim communities, with a considerable number of the marriages occurring between couples of the ages 15–19 years. Under an arrangement of law, Sect. 15 of the Marriage Registration Ordinance, Act No. 18 of 1995, [116] states that a marriage would be unlawful unless both parties were above eighteen years of age. the legal framework allows, Sect. 22 of the same Ordinance, as amended by Act No. 12 of [116] provides exceptions to this Given parental consent, a minor may be married. The parent's consent may be given by the father; if he is dead or incapacitated, then the mother; if both are not present, an identified guardian or the District Court may at times give the consent. Theoretically, these safeguards could be operated towards curbing early marriage, but in actual practice, they have become a publicity for legitimization and continuation of early marriage.

With regard to the level of education it reported that the majority of 48.6% had passed up to grades 6–10. 43.9% had passed the GCE Ordinary Level, and only 5.6% passed the GCE Advanced Level. Among the respondents, the majority of them were unemployed and only 10.3% of them were employed. Of the employed respondents, 4.7% were engaged in retail sales, 4.7% were garment workers and 0.9% were in the healthcare industry. The table below provides the specific profile of the respondents.

#### Descriptive statistics

As the discuss in the previous sections showed, a Likert scale question questionnaire for each variable upon which to rate based on their experience, in scale 1–5 as 1: Strongly Disagree, 2: Disagree, 3: Neutral, 4: Agree, and 5: Strongly Agree, was distributed to the respondents.

#### Social factors

Fig. 4 illustrates the distribution of responses on social factors. The statements “My family encouraged me to continue my education after pregnancy” and “My community supported my efforts to pursue education after pregnancy” both had a mean score of 2.31, with standard deviations of 0.92 and 0.69 respectively. Both statements had a mode of 2 as well. The statement “I did not face stigma or discrimination at the school after becoming pregnant” returned a mean of 2.05 (SD = 0.90) with a mode of 2, while the statement “Teachers and school staff treated me fairly after my pregnancy” reported a mean of 1.99 (SD = 0.84), with a mode of 2.

#### Access to education

Fig. 5 illustrates the distribution of responses on access to education. Under the variable “Access to Education”, the statement “My school allowed me to continue education after pregnancy” reported a mean of 1.87 (SD = 1.00). The statement “Household income was sufficient to access education” returned a mean of 2.05 (SD = 0.77) while “The school I attended was within a reasonable distance from my home” reported a mean of 2.24 (SD = 0.93) and “I had reliable transportation to access education after pregnancy” reported a mean of 2.07 (SD = 0.96). All four statements reported a mode of 2.

#### Access to healthcare

Fig. 6 displays the distribution of responses on access to healthcare. In terms “Access to Healthcare”, the statement “I was treated fairly and respectfully during and after pregnancy by healthcare providers” reported a mean of 4.19 (SD = 0.84) with a mode value of 4. The statement “I had adequate financial support to access healthcare facilities” had a mean value of 2.62, standard deviation of 0.97 with a mode of 2. Third statement “The healthcare services I needed were within a reasonable distance from my home” returned a mean of 3.40 (SD = 1.08) with a mode of 4. The final statement “I had reliable transportation to access healthcare after pregnancy” returned a mean of 2.91 with a standard deviation of 1.16 and a mode of 4.

#### Educational attainment

Fig. 7 presents the distribution of responses on educational attainment. Similar to other variables, educational attainment also had four statement to determine the impact adolescent pregnancy caused on education of young mothers. The first statement “My pregnancy has made it harder for me to concentrate on my studies” reported a mean of 4.31 (SD = 0.88), with a mode of 5. The statement “I have had to make sacrifices in my studies to manage my pregnancy” reported a mean of 4.21 (SD = 0.69) and “My motivation to pursue education decreased after becoming pregnant” returned mean of 3.75 (SD = 0.96). Both two and three statements had the same mode value of 4. The final statement regarding the educational attainment, “Pregnancy was the main reason I dropped out of school” had a mean value of 3.91 (SD = 1.10) with a mode of 4.

#### Employment outcomes

Fig. 8 presents the distribution of responses on employment outcomes. The first statement to measure the impact of employment outcomes, “I found it difficult to find a job due to the lack of educational attainment” returned a mean of 4.12 (SD = 0.76). The statement “My pregnancy impacted my ability to complete required education for better jobs” reported a mean of 4.11 with a standard deviation of 0.80, while the statement “I experienced discrimination in the job market because of my pregnancy” reported a mean of 4.05 (SD = 0.84). The final statement “The low level of education due to pregnancy, stopped me from progressing in career” returned a mean of 3.93 with a standard deviation of 0.82. All four statements regarding employment outcomes shared a mode of 4.

### Measurement model

#### Outer model analysis

In conducting measurement model assessment, first check the indicator reliability through consideration of the outer loading. Whereas all items recorded outer loadings above the recommended 0.40 in norm, the item AH 01 recorded an unacceptable low outer loading of 0.319. This, therefore, rendered it fit for elimination from the model in order to improve the general construct reliability and validity [117].

The Cronbach’s alpha values ranged from 0.666 to 0.781 across all constructs. The lowest Cronbach’s alpha (0.666) was recorded for the Access to Healthcare construct. According to [118, 119], this remains within the acceptable threshold 0f 0.60 to 0.70 for exploratory studies. Composite reliability scores were from 0.807 to 0.858, higher than the cut-off and supporting the internal consistency reliability of the model. Also, the Average Variance Extracted (AVE) scores for constructs were between 0.593 and 0.602, higher than the 0.50 cut-off value [117]. The results of these validity and reliability checks are presented in the following tables (Table 3).

**Table 3:** Results of reliability and convergent validity tests

Construct	Items	Factor Loadings	Cronbach’s Alpha	Composite Reliability	AVE
Social Factors	SF 01	0.761	0.769	0.85	0.587
	SF 02	0.727			
	SF 03	0.797			
	SF 04	0.778			
Access to Education	AE 01	0.815	0.774	0.853	0.593
	AE 02	0.814			
	AE 03	0.715			
Access to Healthcare	AE 04	0.733	0.666	0.807	0.583
	AH 02	0.774			
	AH 03	0.762			
	AH 04	0.755			
Educational Attainment	EA 01	0.753	0.772	0.854	0.594
	EA 02	0.765			
	EA 03	0.776			
	EA 04	0.789			
Employment Outcomes	EO 01	0.775	0.781	0.858	0.602
	EO 02	0.788			
	EO 03	0.821			
	EO 04	0.716			

Source: Author illustrated based on Smart-PLS output

Having established the measurement model to be reliable and valid, the findings are interpreted below within research objectives of the study:

- Objective 01—To measure the impact of social factors on the educational attainment of teenage mothers: Results displayed high measurement reliability from all items with Cronbach's alpha of 0.769, composite reliability of 0.850 and AVE of 0.587.
- Objective 02—To quantify the effect of education access on the education level of adolescent mothers: Results indicated high measurement reliability from all items with Cronbach's alpha of 0.774, composite reliability of 0.853 and AVE of 0.593.
- Objective 03—Measure the effect of access to healthcare on the education level of teenage mothers: The construct after refinement exhibited acceptable reliability from each item with Cronbach's alpha of 0.666, composite reliability of 0.807 and AVE of 0.583.
- Objective 04—To measure the impact of educational attainment of adolescent mothers on their employment outcomes: Both constructs returned strong reliability and convergent validity. Educational attainment showed a Cronbach’s alpha of 0.772, composite reliability of 0.854 and AVE of 0.594, while employment outcomes showed a Cronbach’s alpha of 0.781, composite reliability of 0.858 and AVE of 0.602.

To assess the discriminant validity of the constructs, this research employed the Fornell-Larcker criterion. As put by Fornell and Larcker [117] the square root of AVE of a construct must be higher than its highest correlation with the other inter-constructs. As we observe from the table below (Table 4), all the diagonal values exceed the respective correlation between the other constructs.

**Table 4:** Results of Fornell-Larcker Criterion

	**AE**	**AH**	**EA**	**EO**	**SF**
AE	**0.770**				
AH	0.511	**0.763**			
EA	−0.716	−0.56	**0.771**		
EO	−0.757	−0.554	0.716	**0.776**	
SF	0.728	0.428	−0.643	−0.716	**0.766**

To further validate the model, we look at the Heterotrait-Monotrait ratio (HTMT). This is a more sophisticated way to assess discriminant validity, especially when the constructs are quite similar [120]. The table below (Table 5) shows the results we got from the HTMT test.

**Table 5:** Results of Heterotrait-Monotrait ratio

	**AE**	**AH**	**EA**	**EO**	**SF**
AE					
AH	0.633				
EA	0.905	0.731			
EO	0.958	0.692	0.903		
SF	0.926	0.517	0.808	0.913	

Usually, a threshold of 0.90 or above is considered appropriate to ensure discriminant validity. Yet we found some HTMT values in this study to have surpassed the limit deemed acceptable. While all values between Access to Healthcare and other constructs remained below the threshold, five somewhat concerning values did surface: AE–EA (0.905), AE–EO (0.958), AE–SF (0.926), EA–EO (0.903), and EO–SF (0.913). These elevated values raise potential issues regarding discriminant validity among these constructs. Despite this, we decided to keep the constructs for further analysis, taking into account the results from the Fornell-Larcker criterion, as well as the acceptable reliability and AVE values we observed.

#### Structural model

**Fig. 9:**
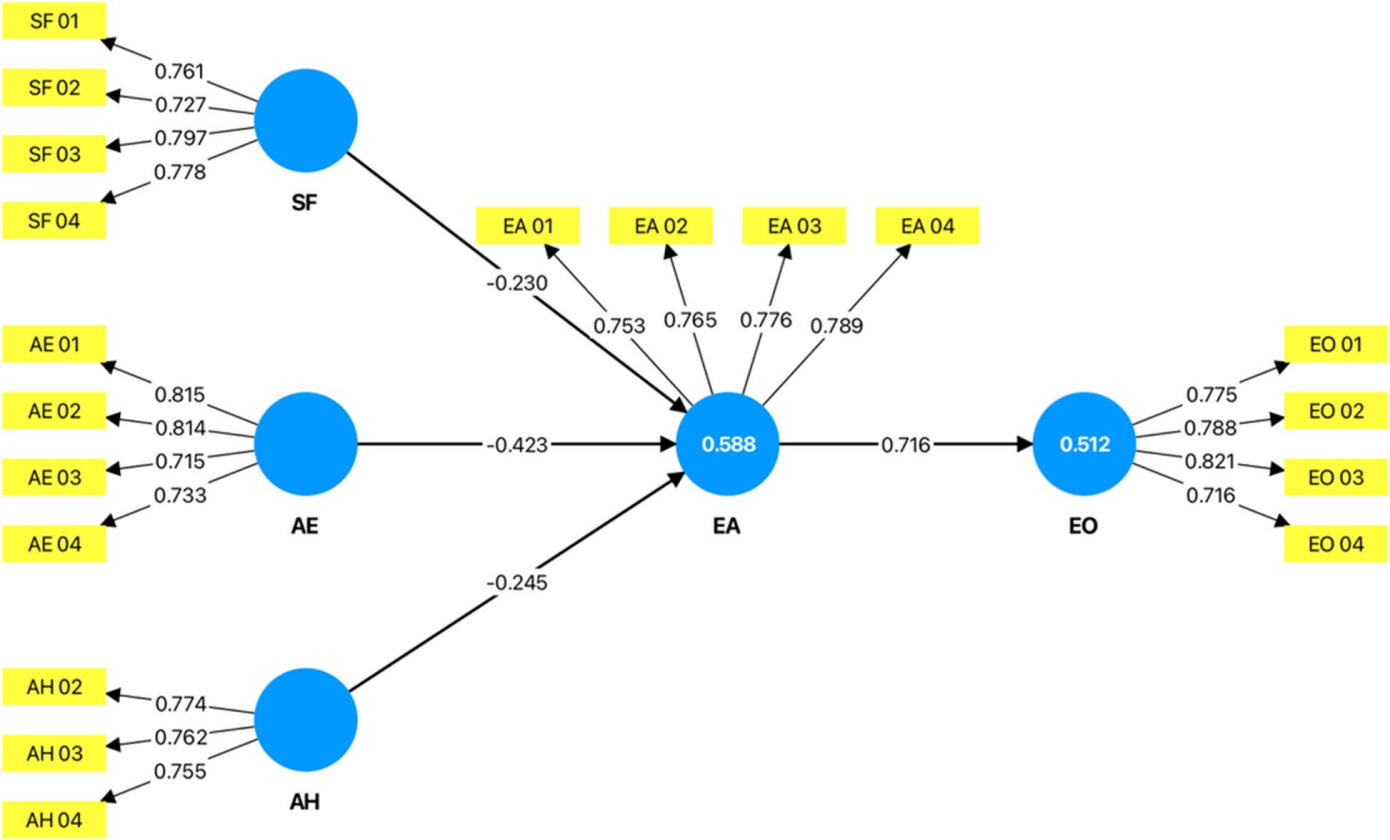
Smart-PLS graphical output of the model. Source: Extracted from Smart-PLS

To examine the relationships between latent variables and test the hypothesis model, inner model analysis was conducted in SmartPLS adopting the bootstrapping procedure with 10,000 sub samples at 5% significance level and generated path coefficients, t-statistics, and p-values to evaluate the significance of hypothesized direct relationships between constructs (Fig. 9). R^2^ values were also examined to assess the explanatory power of endogenous variables used in the model. In order to study the indirect impacts of social variables, access to healthcare and access to education on employment outcomes, mediation analysis was also done with the mediating variable being education.

#### Model evaluation

Using important measurement, such as coefficient of determination (R2) and model fit index (SRMR), robustness and strength of the structural model have been evaluated. R2 represents the proportion of the variance in the endogenous constructs explained by the model (Table 6).

**Table 6:** Results of R-square analysis

Construct	R-Square	R-Square Adjusted
EA	0.588	0.576
EO	0.512	0.507

In this model, Educational Attainment (EA) and Employment Outcomes (EO) both had R2 values of 0.588 and 0.512 respectively, demonstrating moderate level of explanatory power by its predictors.

The Standardized Root Mean Square Residual (SRMR) was also computed to verify the global model fit. The SRMR value obtained for this study was 0.103, which is slightly above this cut-off of 0.08. That global model fit measures such as SRMR are not primary measures for model evaluation in PLS-SEM.

#### Inner model analysis

The results showed that each of the four hypothesized connections was significant at a statistical level (Table 7). That is, the connection between Employment Outcomes (EO) and Educational Attainment (EA) was the strongest with a β of 0.716, t of 10.705 and a p value of 0.005. Connections from Social Factors (SF), Access to Education (AE) and Access to Healthcare (AH) to Educational Attainment were significant but in the negative direction. This is because of the measurement scales of the model, where high SF, AE, and AH have greater support or access and high EA stands for more disturbance in education. Under multicollinearity, VIF was far below 5 for all values in this model, which means there is no serious multicollinearity.

**Table 7:** Results of hypothesis and VIF testing

	**O**	**M**	**STDEV**	**T Statistics**	*P* Values	**VIF**	**Hypothesis**
SF—> EA	−0.230	−0.232	0.083	2.788	0.005	2.212	**Accepted**
AE—> EA	−0.423	−0.415	0.103	4.116	≤ 0.001^*^	2.452	**Accepted**
AH—> EA	−0.245	−0.251	0.067	3.635	≤ 0.001^*^	1.401	**Accepted**
EA—> EO	0.716	0.713	0.067	10.705	≤ 0.001^*^	1.000	**Accepted**

^*^indicates high significance (*P* ≤ 0.001); O: Original Sample; M: Sample Mean; STDEV: Standard Deviation

Findings of the interconnected model strongly validated the four hypotheses set out in the research. Findings shows that a significant negative association between Social Factors (SF) and Educational Attainment (EA) with β = −0.230, t = 2.788, and p = 0.005. The negative relationship indicates greater social support hinders disruption to education among teenage mothers. This supports previous literature which points out that supported teenage mothers those offered financial aid, stationery, and counseling experience lower disruptions in schooling and higher educational attainment than without support [121].

A significant negative connection between Access to Education (AE) and Educational Attainment (EA) was also discovered confirming the second hypothesis. A range of statistics was recorded whereby the relationship produced a β value of −0.423 and t value of 4.116 with p value lower than 0.001, signifying the less interruption caused by teenage pregnancy with education as the education access improves. Prior literature discusses such results as well indicating that the increased access to education improves the level of education among teenage mothers (Nkosi and Pretorius, 2019).

Likewise, the outcomes helped substantiated the supporting findings for hypothesis 03. The correlation of Access to Healthcare (AH) to Educational Attainment (EA) showed a statistically significant negative relationship with β = −0.245, t = 3.635, p < 0.001. This shows that lower maternal healthcare access tends to lessen the adverse effects of healthcare on education. These results are consistent with [122] which demonstrates that the availability of financial, transportation, and proximity to other relevant infrastructural aids influence adolescent mothers’ utilization of maternal health services that affect their enablement to continue schooling after pregnancy.

The relationship between Educational Attainment (EA) and Employment Outcomes (EO), which explains the fourth hypothesis, was found to have significant positive relationship with β value of 0.716, t value of 10.705 and p value less than 0.001. Because higher values of EA represent more disruption on education and higher values of EO represent poorer employment outcomes. These results indicate that low educational attainment is associated with poorer labor market results, aligning with the existing literature [28].

Overall, the structural model findings validate the hypotheses of the study and indicate that improved social conditions, access to school and access to medical service considerably reduce disruption in education caused by adolescent pregnancy in schooling which ultimately play a critical role in shaping teenage mothers’ employment outcomes.

#### Mediation analysis

To examine the indirect effects in the structural model more, a mediation analysis was conducted (Table 8) to test whether Educational Attainment (EA) is a mediator of relationships between Social Factors (SF), Access to Education (AE), and Access to Healthcare (AH) and Employment Outcomes (EO). Medication was tested using the significance of indirect effects and their respective path coefficients.

**Table 8:** Results of mediation analysis

	**O**	**M**	**STDEV**	**T Statistics**	*P* Values
SF—> EO	−0.165	−0.166	0.063	2.603	0.009
AE—> EO	−0.302	−0.299	0.086	3.531	≤ 0.001^*^
AH—> EO	−0.175	−0.178	0.046	3.781	≤ 0.001^*^

^*^ indicates high significance (P ≤ 0.001); O: Original Sample; M: Sample Mean; STDEV: Standard Deviation

Mediation analysis outcomes showed that Educational Attainment (EA) significantly mediates the effects of social Factors, Access to Education, and Access to Healthcare on Employment Outcomes. Three indirect effects were significant as SF → EO: β = −0.165, AE → EO:β = −0.302 and AH → EO: β = −0.175. These findings illustrate that enhanced social support, education access, and healthcare access reduce the school disruption caused by teen pregnancy, enhancing the job prospects of teen mothers.

## Discussion

The findings of this study demonstrate various degree of alignment with the initial objectives and hypotheses, offering meaningful insights into how adolescent pregnancy affects educational attainment and future employment prospects, particularly within the socioeconomically challenged district of Batticaloa, Sri Lanka.

Based on the study, the study sample and adolescent mothers included 107 respondents, 61.7% of WHOm were aged 10 to 18 years old during the study and 88.8% of WHOm reported experiencing pregnancies aged 16 to 19 years old, while United Nations Children's Fund, 2024 state that, with the later age range being universal parlance for pregnancies by teen girls that are documented primarily in mid-to-late adolescence [36] Ethnic breakdown shows that, the sample was consist with largely Tamil (54.2%) and Muslim (29.9%), which is a reflection of the population composition of Batticaloa District [65].

In accordance with the first objective of the study, it has been discovered that socioeconomic factors are a strong predictor of academic performance among adolescent mothers. There was a significant negative relationship between socioeconomic factors and academic performance (t = 2.788, p = 0.005). In the same line with research by [51] and [123] it is evident that poverty, early marriage, and lack of family support are main factors for dropout from schools among teenage mothers. In this study, 89.7% of the subjects were out of work, while over 48% of them had not progressed past Grade 10, showing that lower socioeconomic status directly curtails education opportunity. The study concurs with previous studies done by [49] and [124] indicated that economic instability combined with family and early marriage result in the abandonment of education. These findings also agree with a study by [59] that established that economic vulnerability has a direct connection to family income and school performance leading to school dropout among pregnant adolescents.

The research findings indicated that how restricted educational opportunities affect adolescent mothers'pursuit of educational goals. The statistical analysis demonstrated that educational access maintained a notably negative effect on educational success because as the opportunities disappear the achievement outcomes decrease (β = −0.415, t = 4.116, p < 0.001). Research by [125] supports findings which show adolescent mothers have to drop out of school because of social prejudices and institutional biases. The present study is that only 5.6% of the respondents had reached the G.C.E. A/L level despite being of the correct age. This finding reveals a systemic failure to create an enabling environment for continuity in education after pregnancy.

In addition, 61.7% of the sample population were aged between 10 and 18 years at the time of study within the school-going age but most had already left the education system as a result of early motherhood. This clearly indicates that adolescent pregnancy remains a significant disruptor of educational trajectories in Sri Lanka. The research findings receive support from [20] study which demonstrates entrenched sociocultural norms as fundamental factors driving the school dropout of young mothers. According to Akella and Jordan [20] teenage pregnancy often faces moral condemnation in traditional societies that leads young mothers to experience social exclusion and inner shame and subsequently drop out of education.

The research data indicated access to healthcare services proved to be a major statistical predictor variable for educational achievement (β = −0.251, t = 3.635, p < 0.001). Research findings from Rajarata Pregnancy cohort in Sri Lanka Agampodi et al., 2022 [22] and Rural Lao [126] supports the discovery that limited adolescent-friendly health services link to adverse maternal outcomes and educational interruptions. Moreover, research finding found that In Batticaloa, 42% of participants reported inadequate reproductive healthcare which restricted their ability to handle both academic work and becoming mothers. A study by Stanger Hall [60] demonstrated that Medicaid family planning waivers combined with healthcare access produce measurable effects on teen pregnancy rates that shape girls'paths through education. Similarly, Agampodi Wickramasinghe [22] demonstrate, Sri Lanka's rural areas face higher economic burdens and cultural expectations on adolescent girls which increase their barriers to health services.

Research data analysis confirmed that education levels create straightforward employment benefits as they have a very high positive correlation (β = 0.713, t = 10.705, p < 0.001) with employment opportunities and educational attainment. According to the research data, interviewee employment rates attained 10.3% when they were employed only in minimum-wage garment and retail industries. Results of the study affirm [127] and [78] research to show how adolescent pregnancy intervenes in schooling and creates barriers to stable skilled employment. The implication is that adolescent mothers are likely to obtain informal or small-or-no-skills employment rather thanprofessional work. This has also been attested by [128] that depict teen mothers are more likely to end up in low-skilled and low-paid jobsdue to their few qualifications. With insufficient qualifications for higher-skilled employment adolescent mothers either end up being joblessor stuck in informal low-paid work. Social exclusion and poverty with dependency enact a robust cycle that calls for unique intervention programs. In conclusion, These findings support all four hypotheses of this study, indicating that poor social factors, access to education andaccess to healthcare reduce the level of education of adolescent mothers, ultimately impacting their employment opportunities.

### Limitation

The research is mainly concerned with the effects of teenage pregnancy on these young mothers’ education and job prospects in Batticaloa District Sri Lanka. As the analysis comes from teenage mothers in the Batticaloa District only, it is not clear if the conclusions can be used for the whole country. Thus, it would be good to assess in different areas and check the outcomes in various socio-cultural and economic settings. Moreover, it does not consider perspectives of other key stakeholders such as teachers and parents view the same issue, which could add more detailed information on the problem. More investigation could be done to consider these other aspects and aid the creation of better support systems for teenage mothers.

## Conclusion

This study investigates how adolescent pregnancy affects schooling and possible employments of young mothers in Batticaloa, Sri Lanka. They are reported to be facing some forms of socio-economic constraints, stigma, little public health and education, and societal expectations around these issues, which cause very severe burdens on them.

In the end, these factors have further extended the poverty cycle and negated opportunities for personal and professional development. The research has identified gaps in the institutional support systems, which need to be addressed quite urgently. Policies relating to the changing context of negative impacts from adolescent pregnancies should include access to comprehensive sexual and reproductive health education, vocational training, affordable childcare, and mental health counseling.

A policy that involves several sectors should be created to help adolescent mothers in Sri Lanka. Bridge courses, night schools, and web-based lessons should be part of the program for teen parents in New Zealand’s Teen Parent Units and alternative learning systems in the Philippines (Education Review Office, 2018; Republic of the Philippines Department of Education, 2018). Clear, stigma-free re-entry policies must be enacted, as in Malawi and Tanzania [129]. Vocational training and mentorship programs, like Kenya's"Yes Youth Can,"can enhance employment opportunities, while social-based awareness initiatives can offset stigma [130]. Last but not least, an adolescent-specific reproductive health law as Ethiopia did is required to ensure access to confidential, comprehensive services [77, 131]. All these measures combined can allow young mothers to pursue education and work without hindrance.

It is necessary but not exhaustive to include community-based initiatives and culturally sensitive programs for mitigating stigma and facilitating return to education and employment levels. It will take an integrated, evidence-based approach to last for stakeholders to allow adolescent mothers to have a better chance of breaking out of critical chains of disadvantages while moving forward with socioeconomic development and gender equity in Batticaloa and similar communities. Future studies should focus on the impacts in the long run and identify successful interventions to develop a sustainable path towards furthering adolescent mothers'welfare and empowerment.

## Supplementary Information

Supplementary Material 1

Supplementary Material 2

## Data Availability

Yes, I have research data to declare. The data that support the findings of this study are available from the corresponding author upon reasonable request.

## Abbreviations

OECD: Organization for Economic Co-operation and Development
WHO: World Health Organization
AYFHS: Adolescent and Youth-Friendly Health Services
SLIIT: Sri Lanka Institute of Information Technology
A/L: Advanced Level
O/L: Ordinary Level
