# Supplementary material for: Socioeconomic impacts of adolescent pregnancy on education and future employment in Batticaloa District, Sri Lanka
Source: BMC Public Health. 2025 Oct 1;25:3262. doi: 10.1186/s12889-025-24108-x (PMC12487213; doi:10.1186/s12889-025-24108-x)
Supplement: Supplementary file 2 — Supplementary Material 2 [file 12889_2025_24108_MOESM2_ESM.docx]

**Sri Lanka Institute of Information Technology**


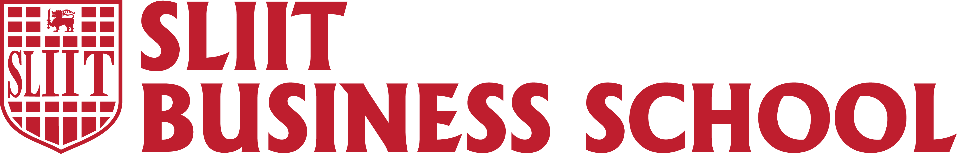


**Impact of Adolescent Pregnancy on Education and Future Employment Prospects in Sri Lanka: Findings of the Batticaloa Teenage Pregnancy Cohort**

**Ethical Statement and Consent:**

Thank you for participating in this research study on the "Impact of Adolescent Pregnancy on Education and Future Employment Prospects in Sri Lanka." Before you proceed, please read the following information carefully:

**Purpose of the Study**:

- This study aims to understand the effects of adolescent pregnancy on education and future employment opportunities in Sri Lanka.

**Voluntary Participation:**

- Your participation in this study is entirely voluntary. You may choose to withdraw at any time without any consequences.

**Confidentiality:**

- All the information you provide will be kept strictly confidential. Your responses will be anonymized and will not be linked to your identity in any way.

**Data Usage**:

- The data collected will be used solely for academic purposes and will be stored securely. Results may be published in academic journals, but individual responses will not be identifiable.

**Risks and Benefits:**

- There are no foreseeable risks associated with participation in this study. While there are no direct benefits to you, your responses will contribute valuable insights to the research.

By continuing with this questionnaire, you acknowledge that you have read and understood the above information and consent to participate in this study.

**Respondent Signature: ………………………**

**Section 01: Demographic Information**

Age:

Age at the Time of Pregnancy:

Ethnicity:

Marital Status (Single / Married / Divorced or Separated / Other):

Level of Education (No formal education / Grade 1-5 / Grade 6-10 / GCE Ordinary Level / GCE Advanced Level):

Employment Status (Employed / Not employed):

Occupation:

**Section 02: Social Factors**

| **Statement** | **Scale** | **References** |
| --- | --- | --- |
| My family encouraged me to continue my education after pregnancy | 1: Strongly Disagree  2: Disagree  3: Neutral  4: Agree  5: Strongly Agree | ([Yurdakul, 2018](#_ENREF_10)) |
| My community supported my efforts to pursue education after pregnancy |  | ([Nsanzabera et al., 2024](#_ENREF_5)) |
| I faced stigma or discrimination at the school after becoming pregnant |  | ([Nsanzabera et al., 2024](#_ENREF_5)) |
| Teachers and school staff treated me fairly after my pregnancy |  | ([Nsanzabera et al., 2024](#_ENREF_5)) |

**Section 03: Access to Education**

| **Statement** | **Scale** | **References** |
| --- | --- | --- |
| My school allowed me to continue education after pregnancy | 1: Strongly Disagree  2: Disagree  3: Neutral  4: Agree  5: Strongly Agree | ([Gyan, 2013](#_ENREF_4)) |
| Household income was sufficient to access education |  | ([Shaningwa, 2007](#_ENREF_7)) |
| The school I attended was within a reasonable distance from my home |  | ([Shaningwa, 2007](#_ENREF_7)) |
| I had reliable transportation to access education after pregnancy |  | ([Shaningwa, 2007](#_ENREF_7)) |

**Section 04: Access to Healthcare**

| **Statement** | **Scale** | **References** |
| --- | --- | --- |
| I was treated fairly and respectfully during and after pregnancy by healthcare providers | 1: Strongly Disagree  2: Disagree  3: Neutral  4: Agree  5: Strongly Agree | ([Alam et al., 2016](#_ENREF_1)) |
| I had adequate financial support to access healthcare facilities |  | ([Vongxay et al., 2019](#_ENREF_9)) |
| The healthcare services I needed were within a reasonable distance from my home |  | ([Vongxay et al., 2019](#_ENREF_9)) |
| I had reliable transportation to access healthcare after pregnancy |  | ([Alam et al., 2016](#_ENREF_1)) |

**Section 04: Impact of Pregnancy on Educational Attainment**

| **Statement** | **Scale** | **References** |
| --- | --- | --- |
| My pregnancy has made it harder for me to concentrate on my studies | 1: Strongly Disagree  2: Disagree  3: Neutral  4: Agree  5: Strongly Agree | ([Otegbayo et al., 2023](#_ENREF_6)) |
| I have had to make sacrifices in my studies to manage my pregnancy |  | ([Bałanda et al., 2020](#_ENREF_2)) |
| My motivation to pursue education decreased after becoming pregnant |  | ([Daisy et al., 2022](#_ENREF_3)) |
| Pregnancy was the main reason I dropped out of school |  | ([Gyan, 2013](#_ENREF_4)) |

| **Statement** | **Scale** | **References** |
| --- | --- | --- |
| I found it difficult to find a job due to the lack of educational attainment | 1: Strongly Disagree  2: Disagree  3: Neutral  4: Agree  5: Strongly Agree | ([UNFPA, 2019](#_ENREF_8)) |
| My pregnancy impacted my ability to complete required education for better jobs |  | ([UNFPA, 2019](#_ENREF_8)) |
| I experienced discrimination in the job market because of my pregnancy |  | ([UNFPA, 2019](#_ENREF_8)) |
| The low level of education due to pregnancy, stopped me from progressing in the job |  | ([UNFPA, 2019](#_ENREF_8)) |

**Section 05: Impact of Pregnancy on Employment Outcomes**

Alam, N., Chowdhury, M. E., Kouanda, S., Seppey, M., Alam, A., Savadogo, J. R., Sia, D. & Fournier, P. (2016). "The role of transportation to access maternal care services for women in rural Bangladesh and Burkina Faso: A mixed methods study". International Journal of Gynecology & Obstetrics, 135, pp. S45-S50.Available at: <https://doi.org/10.1016/j.ijgo.2016.09.003>

) Accessed: 2025/04/28].

Bałanda, -. B., Agnieszka, Pilewska, -. K., Anna B., Łepecka, -. K., Celina, Stadnicka, G. & Dobrowolska, B. (2020). "Attitudes of Teenage Mothers towards Pregnancy and Childbirth". International Journal of Environmental Research and Public Health, 17, pp.Available at: <https://doi.org/10.3390/ijerph17041411>) Accessed].

Daisy, C., Gladys, K. & James, K. (2022). "Influence of Low Self-Esteem Due To Teenage Pregnancy on Educational Performance among Pregnant Teenagers in Secondary Schools in Narok County". East African Journal of Humanities and Social Sciences, 01, pp.Available at: <https://www.utafitionline.com/index.php/eajhssISSN> ) Accessed].

Gyan, C. (2013). "Effects of teenage pregnancy on the educational attainment of girls at Chorkor, a suburb of Accra". Journal of Educational and Social Research, 3, pp.Available at: Accessed: 12th June 2024].

Nsanzabera, F., Evangeline, I., Emmanuel, N., Alexis, M., Bosco, N. J., Aimable, M. & and Nkurikiyimana, F. (2024). "The community support and its role in adolescent pregnancy and school reintegration in rural Rwanda: a mixed-methods study". Global Health Action, 17, pp. 2436716.Available at: <https://doi.org/10.1080/16549716.2024.2436716>

) Accessed].

Otegbayo, B. E., Omar, N., Danaee, M., Mohajer, S. & Aghamohamadi, N. (2023). "Impact of individual and environmental factors on academic performance of pregnant adolescent". BMC Women's Health, 23, pp. 383.Available at: Accessed: 29th July 2024].

Shaningwa, L. (2007). "The educationally-related challenges faced by teenage mothers on returning to school : a Namibian case study". pp.Available at: Accessed].

UNFPA 2019. Methodology for assessing the economic impact of adolescent pregnancy and early motherhood in latin american and caribbean countries. *United Nations Population*

*Fund - Latin American and the Caribbean Regional Office. Panama. 2019.*

Vongxay, V., Albers, F., Thongmixay, S., Thongsombath, M., Broerse, J. E. W., Sychareun, V. & Essink, D. R. (2019). "Sexual and reproductive health literacy of school adolescents in Lao PDR". PLOS ONE, 14, pp. e0209675.Available at: <https://doi.org/10.1371/journal.pone.0209675>) Accessed].

Yurdakul, M. (2018). "Perceived social support in pregnant adolescents in Mersin area in Turkey". PubMed, pp.Available at: DOI: 10.12669/pjms.341.14221) Accessed: 2018 Jan].

**References**
